# Zoledronate Therapy for the Pathological Humeral Fracture in Polyostotic Fibrous Dysplasia: A Case Report

**DOI:** 10.14740/jocmr2318w

**Published:** 2015-09-25

**Authors:** Ikko Ohno, Chikahisa Higuchi

**Affiliations:** a Department of Orthopaedics, Osaka Bay Central Hospital, 1-8-30 Chikko, Minato Ward, Osaka City, 552-0021, Japan; b Osaka Medical Center and Research Institute for Maternal and Child Health, 840 Murodo-cho, Izumi City, 594-1101, Japan

**Keywords:** Fibrous dysplasia, Zoledronate, Pathological fracture

## Abstract

Fibrous dysplasia (FD) of bone is a rare skeletal disease often associated with bone pain, deformities and fractures. The bisphosphonate therapies are reported to be useful for bone pain, but seem to be not suitable for fracture repairs of extremities. This is the first report of zoledronate-induced radiological improvement and long bone fracture union in polyostotic FD. A 30-year-old Japanese female had bilateral shepherd’s crook deformities typical to FD and right pathological femoral fracture and left humeral fracture nonunion. These fractures occurred without major traumas and the humeral fracture was not united for 1 year with conservative therapy. Laboratory blood test results were notable for elevated serum alkaline phosphatase and urine N-terminal cross-linking telopeptide of type I collagen. Her subtrochanteric femoral fracture was percutaneously fixed using Kirschner wires. After surgery, a hip spica cast was applied for 2 months and the orthosis for the next 2 months. Bony union of the femoral fracture was observed 5 months after surgery. Increased bone turnover and typical radiological features suggested that the constant elbow pain was due to both FD itself and humeral nonunion. Considering the possible side effects of zoledronate delaying acute fracture healing, we initiated zoledronate (Zometa^®^; Novartis, Tokyo, Japan) therapy after femoral fracture union. Intravenous zoledronate acid was administered at a dose of 2 mg, along with supplementation of calcium (600 mg/day) and vitamin D (alfacalcidol 0.5 μg/day) to limit the risk of osteomalacia and improve the efficacy of bisphosphonate therapy. The patient’s elbow pain rapidly resolved 1 week after treatment. Second therapy with same dose was performed after 6 months. No recurrence of elbow pain was reported and bony union was diagnosed after 1 year from the first administration. This patient is currently doing well without recurrence of bone pain. She can also walk for a short distance with crutches. We presented the case of an FD patient with persistent elbow pain due to FD itself and nonunion of humeral fracture, which was ameliorated promptly by intravenous zoledronate therapies. This case illustrated the benefit of zoledronate treatment in patients with extensive polyostotic FD and pathological fractures of extremities.

## Introduction

Fibrous dysplasia (FD) of bone is a rare disease often associated with severe clinical outcomes, such as bone pain, bone deformities, and fractures. Histological studies have revealed that this condition is caused by extensive proliferation of fibrous tissue within the bone marrow, due to poorly differentiated mutated osteoblasts. These osteoblastic cells produce an excess amount of interleukin 6, resulting in increased osteoclastic activity, thereby leading to expansile lytic lesions within the fibrous tissue and surrounding normal bone [1]. Bisphosphonates were introduced in the treatment of FD to decrease the increased rate of bone resorption and relieve bone pain. However, bisphosphonates are usually contraindicated in cases of acute fractures because osteoclastic activity is considered to play an important role in fracture repair [2]. We present the case of a patient in whom pain relief and bony union were achieved by administration of zoledronate. Zoledronate has been reported to be effective to facial and cranial bony regions of FD. To the best of our knowledge, this is the first report of zoledronate-induced radiological improvement and extremities’ fracture union in polyostotic FD, accompanied by good clinical improvement.

## Case Report

**Figure 1:**
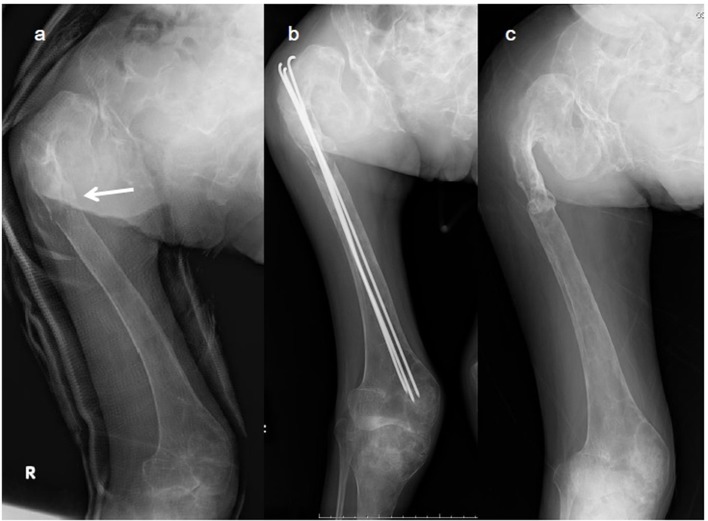
X-ray images of the right femur. (a) Preoperative radiograph of the right femur showing a pathological subtrochanteric femoral fracture (arrow) and a diffuse fibrodysplastic lesion of the entire femur with the typical shepherd’s crook deformity. (b) Femoral fracture was fixed percutaneously with three titanium Kirschner wires through the great trochanter to the distal femoral condyle. (c) Radiograph 1 year after the procedure confirming bony union.

**Figure 2:**
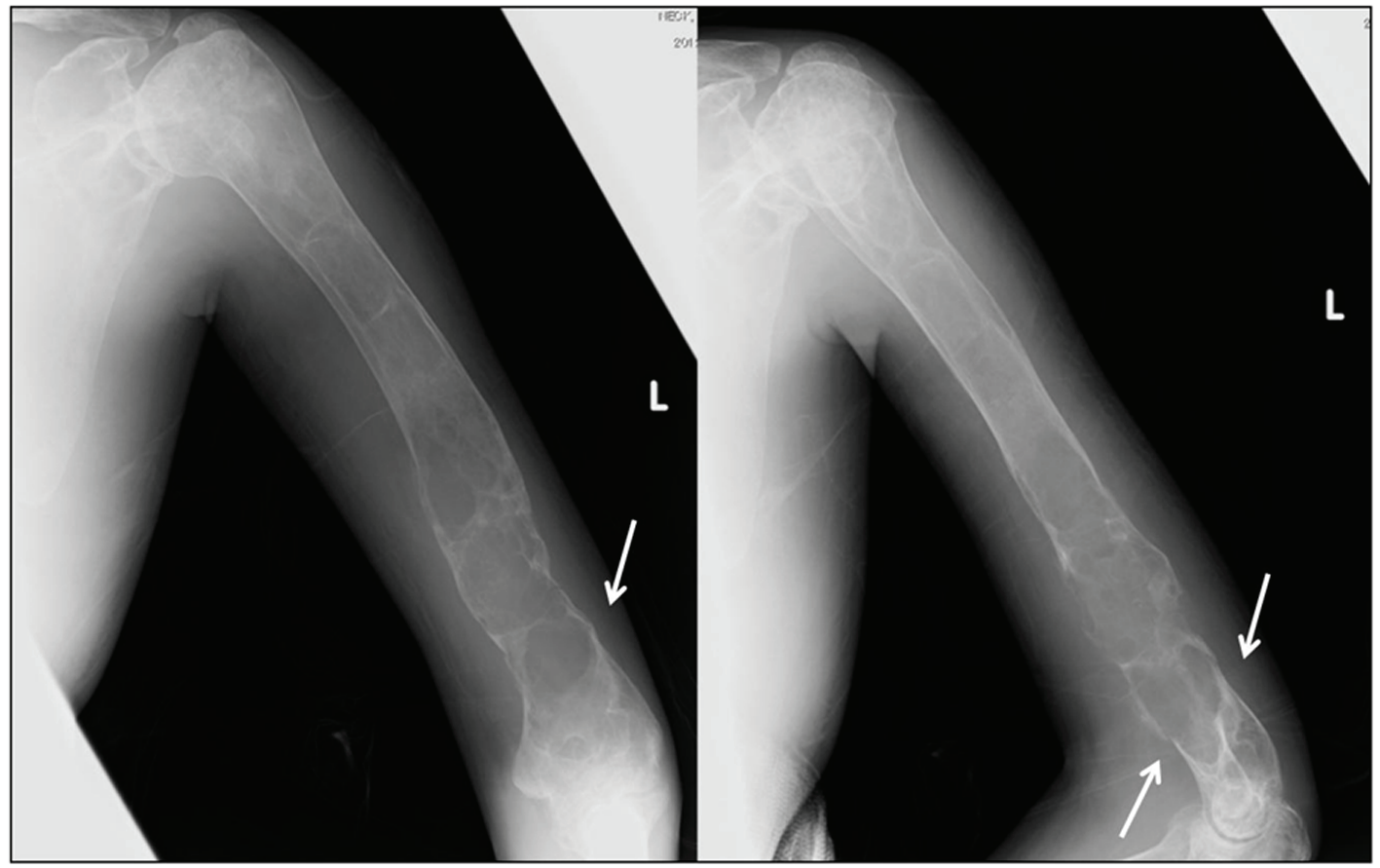
X-ray images of the left humerus at the presentation. Plain radiography shows osteolytic lesions with slightly sclerotic septa in the left humeral shaft and metaphysis. A supracondylar fracture (arrow) is evident.

A 30-year-old Japanese female presented to a local hospital with acute pain in the right thigh after walking a short distance. She did not give any history of major trauma. A pathological right subtrochanteric femoral fracture (Fig. 1a) was detected, and she was referred to our hospital for surgical treatment. She gave the history of mild pain and swelling in her left elbow 1 year ago. She was detected to have a pathological supracondylar humeral fracture (Fig. 2) at the same local hospital and a functional brace was applied as treatment, but this humeral fracture was not united.

At the time of admission, the patient’s height was 119 cm and weight was 40 kg. The patient had severe skeletal deformities and scoliosis. Radiographic skeletal survey revealed thin cortical bone, cystic lesions, and ground-glass appearance of the skull, ribs and almost all skeletal bones, as well as typical bilateral shepherd’s crook deformities of the proximal femurs. These typical radiological features indicated polyostotic FD. The patient had no history of medical interventions for polyostotic FD in her childhood.

Laboratory blood test results were notable for elevated serum alkaline phosphatase (ALP) (2,118 IU/L (reference range, 104 - 338)) and elevated serum bone-specific alkaline phosphatase (BAP) (136 μg/L (reference range, 2.9 - 14.5)) levels. Urine N-terminal cross-linking telopeptide of type I collagen (NTX) was 1,492 nmol bone collagen equivalents/mmol creatinine (nmolBCE/mmolCr) (reference range, 9.3 - 54.3). Serum calcium (8.7 mg/dL (reference range, 8.5 - 10.2)), phosphorus (2.8 mg/dL (reference range, 2.5 - 4.5)), and parathyroid hormone levels (41 pg/mL (reference range, 10 - 65)) were normal. Renal phosphate wasting was not observed (0.54 g/day (reference range, 0.5 - 2.2)). Elevated free thyroid hormone (11.4 pg/mL (reference range, 2.3 - 4.3)) was detected (Table 1).

**Table 1:** Laboratory Blood Test Results at First Admission

Total protein (g/dL)	6.2	Ca (mg/dL)	8.7	WBC	9,800
Albumin (g/dL)	3.7	P (mg/dL)	2.8	RBC	466 × 10^3^
AST (IU/L)	21	Na (mmol/L)	139	Hb	13.1
ALT (IU/L)	17	K (mmol/L)	3.5	Ht	38.3
ALP (IU/L)	2,118	Cl (mmol/L)	101	Plt	27.3 × 10^3^
LDH (IU/L)	126	BUN (mg/dL)	14.5	FT3 (pg/mL)	11.4
PTH (pg/mL)	41	Creatinine (mg/dL)	0.11	FT4 (ng/dL)	4.14
Urine NTX (nmol bone collagen equivalents/mmol creatine)	1,492	Bone alkaline phosphatase (μg/L)	136	Total urine phosphate (g/day)	0.54

The right subtrochanteric femoral fracture was percutaneously fixed using three titanium Kirschner wires that were inserted through the great trochanter to the distal femoral condyle (Fig. 1b). After surgery, a hip spica cast was applied for 2 months, which was followed by the orthosis that spanned from the trunk to the foot for the next 2 months. Bony union of the femoral fracture was observed 5 months after surgery (Fig. 1c). Although the left humeral fracture was initially treated with a functional brace, cortical bone breakdown was clearly observed and bony union was not evident (Fig. 2). Increased bone turnover and the broadened distal humerus metaphysis with thin cortices suggested that the constant elbow pain was partially due to FD itself. Stanton et al recommended internal fixation of the concerned upper extremities in patients with lesions secondary to FD in the upper extremity, who require supportive devices (crutches or canes) for lower extremity conditions [3]. However, we did not perform elastic nail fixation for the humeral fracture because it would not provide rigid fixation and rotational stability due to broad canal. Considering the possible side effects of zoledronate delaying acute fracture healing, we initiated zoledronate (Zometa^®^; Novertis, Tokyo, Japan) therapy 5 months after surgery, when the femoral fracture had united. Written informed consent was obtained from the patient and her family for zoledronate administration because this drug has not been approved in Japan for the treatment of FD. The ethical review board at our hospital approved the prescription of zoledronate in this patient.

**Figure 3:**
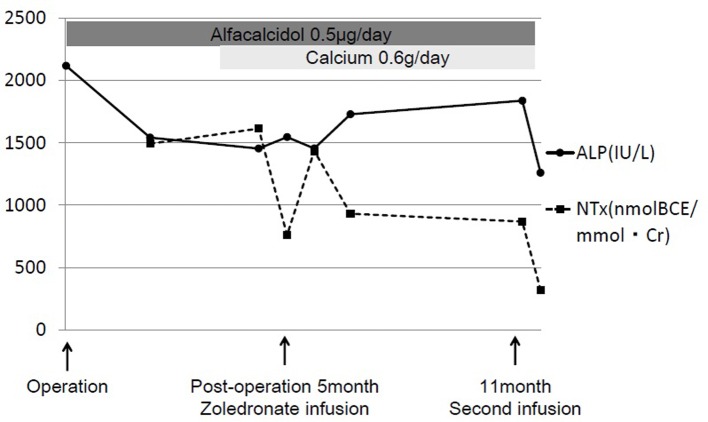
The clinical course of zoledronate therapy. With supplementation including calcium (600 mg/day) and vitamin D (alfacalcidol 0.5 μg/day), 2 mg of intravenous zoledronate (Zometa^®^; Novartis, Tokyo, Japan) was administered at 5 months and 11 months after surgery for the right femoral fracture. Alkaline phosphatase (ALP) levels decreased from 2,118 to 1,366 IU/L and urine N-terminal cross-linking telopeptide of type I collagen (NTX) levels decreased from 1,492 to 319 nmol bone collagen equivalents/mmol creatinine (nmolBCE/mmolCr).

**Figure 4:**
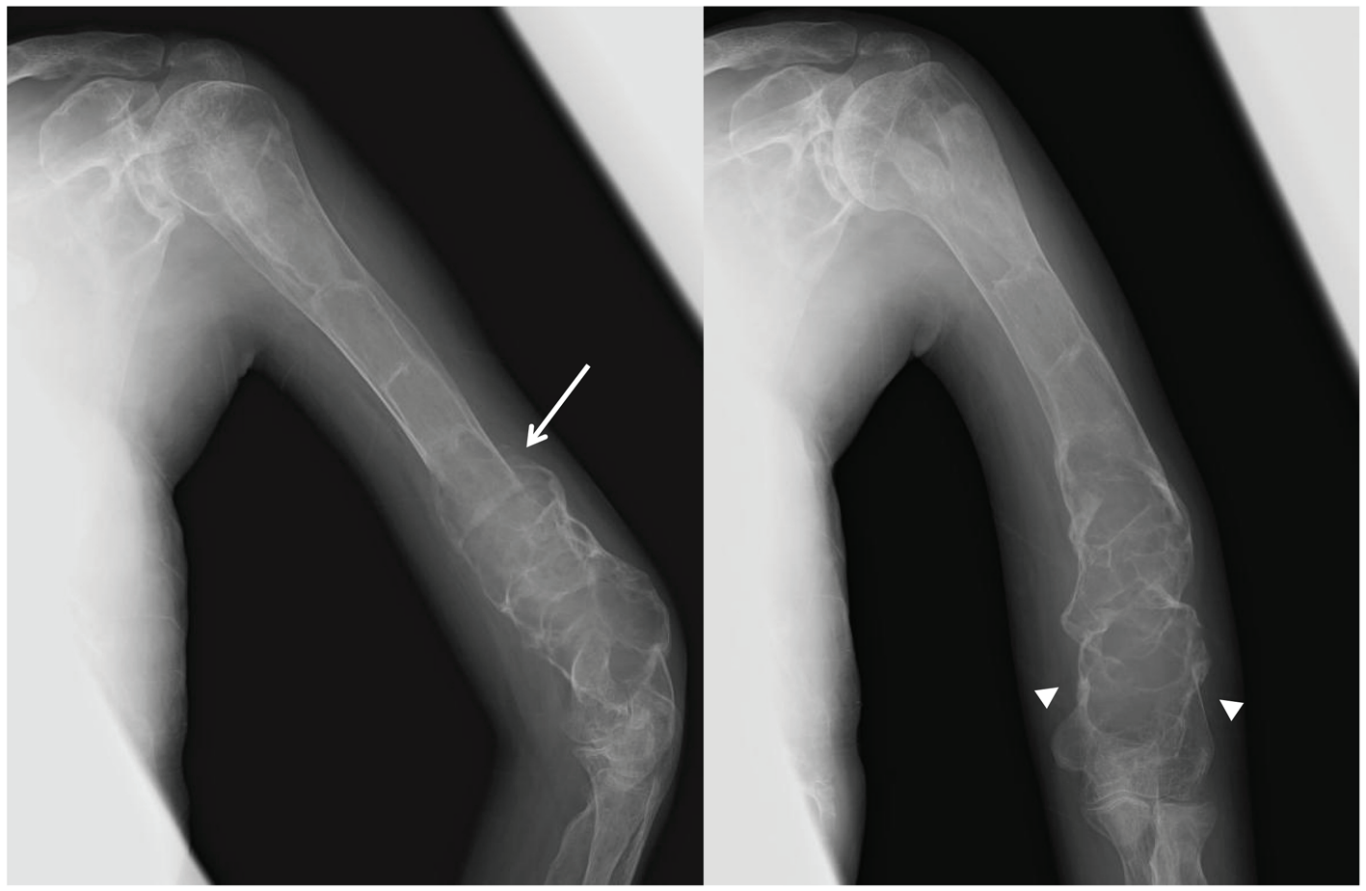
X-ray images of the left humerus after 1 year from the zoledronate administration. Radiological thickening of the humeral shaft cortical bone, progressive ossification in the fibrodysplastic lesion, and periosteal bone formation (arrow) are observed. Fracture lines have completely disappeared (arrow head).

Intravenous zoledronate acid was administered at a dose of 2 mg, along with supplementation of calcium (600 mg/day) and vitamin D (alfacalcidol 0.5 μg/day) to limit the risk of osteomalacia and improve the efficacy of bisphosphonate therapy [4]. The standard protocol of zoledronate therapy for FD had not been published in Japan and her weight was very low, so the dose of zoledronate was determined according to the protocol for treating children with osteogenesis imperfecta, which recommends a dose of 0.05 mg/kg up to 4 mg/day administered over 45 min once every 6 months [5]. The patient’s elbow pain rapidly resolved 1 week after treatment. BAP levels did not change, and NTX levels decreased to 759 nmolBCE/mmolCr. Six months following treatment, no recurrence of elbow pain was reported, but BAP was 179 μg/L and NTX was 867 nmolBCE/mmolCr, both very high compared with the normal standard. A second administration of zoledronate was administered after 6 months. The patient’s ALP fell to 1,366 IU/L, BAP to 135 μg/L, and NTX to 319 nmolBCE/mmolCr (Fig. 3). Radiological thickening of the humeral shaft cortex, progressive ossification of the fibrodysplastic lesion, and periosteal bone formation were observed and bony union was diagnosed after 1 year from the first administration (Fig. 4). This patient is currently doing well without recurrence of bone pain. She can also walk for a short distance with crutches. But we continue this zoledronate therapy at every 6 months intervals due to high bone turnover and multiple osteofibrous regions.

## Discussion

FD of bone is a rare disease that may cause bone pain, fractures, or deformities. Pain was prevalent in the FD population, with 67% patients reported to have pain at FD sites, which is more common in adults than children [6]. Various medications (non-steroidal anti-inflammatory drugs, narcotics, and bisphosphonates) have been prescribed to patients complaining of bone pain. Several research groups have published data on the treatment of FD with bisphosphonates (pamidronate, alendronate, and zoledronate), but these are limited to case reports and patient series. Liens et al described firstly in 1994 a series of 30 patients treated with pamidronate (180 mg) administered at 6-month intervals for three cycles, after which it was administered annually [7]. They formulated the dose and treatment courses in line with the most efficacious treatment protocol for Paget’s disease available at the time of their study. After a mean duration of follow-up of 39 months, they demonstrated improvement in bone pain, decrease in bone turnover, and radiological improvements, with disappearance of osteolytic lesions in approximately 50% of the treated individuals. Chapurlat also reported that pamidronate therapy (180 mg) relieved bone pain and decreased bone resorption with improvements in radiological findings in less than 50% of 62 treated patients [4]. A total of nine patients in their study were switched to zoledronate treatment because of relapse of bone pain and worsening levels of biochemical markers of bone turnover after pamidronate therapy. All these patients had polyostotic FD, and 33% had McCune-Albright syndrome. In this group, five patients who had bone pain when they started zoledronate reported a decrease in the mean pain score. Serum type 1 collagen C-terminal breakdown products levels were decreased, but not significantly. Mansoori et al also reported successful zoledronate treatment of polyostotic FD involving the skull, humerus, and ribs in a 32-year-old man presenting with chronic headache [8]. Intravenous pamidronate (60 mg) was administered to this patient at 6-month intervals for 12 months; however, the patient continued to have severe headache 14 months after treatment initiation. With a switch to 5 mg of zoledronate, the symptoms rapidly resolved 1 week after treatment. One month after treatment with zoledronate, levels of biochemical markers decreased significantly and computed tomography of the head showed dramatic improvement of lesions. On the other hand, Mrabet et al showed successful pamidronate treatment after failure to respond to three infusions (4 mg) of zoledronate administered at 6-month intervals [9]. The reason for these mixed results is not clear; however, partial differences might be related to the condition of FD (increased bone turnover) as well as the absence of the use of phosphate supplements in patients with renal phosphate wasting [4]. There are no current studies showing the efficacy of either pamidronate or zoledronate as first-line therapy for FD. These reports were limited in the treatment of bone pain and no reports presented prescription of zoledronate for the nonunion of extremities’ fractures.

The patient described in this report had polyostotic FD lesions in almost all bones along with a significantly high bone turnover (extremely high NTX and BAP). Therefore, a more potent bisphosphonate (zoledronate) was intravenously administered to her. The general safety profile of intravenous bisphosphonates has been satisfactory. Side effects reported with use of these drugs include transient fever (mostly occurs with the initial infusion), flu-like symptoms, and hypocalcemia [4]. Our patient did not present with any of these symptoms. Considering that osteoclastic activity seems to play an important role in fracture repair, it seems that administration of bisphosphonate therapy immediately after acute fractures should not be recommended because of suppression of bone resorption [2]. To decrease the possibility of side effects of zoledronate delaying fracture healing, we started zoledronate therapy after the femoral had united. Munns et al suggested that in patients with osteogenesis imperfecta, pamidronate is associated with delayed healing of osteotomy sites with periosteal injury in operations, but has no relation to fracture healing time without periosteal avulsion [10]. Because the fixation was performed percutaneously in this patient, the femoral fracture was not surgically exposed and the periosteum was not stripped off. Kiely reported that failed cases of distraction osteogenesis maintained some underlying anabolic activity, and could be treated successfully with bisphosphonate therapy (pamidronate and zoledronate) [11]. It seems that in situations with a pathologically high bone turnover, bisphosphonates would have an excellent effect on osteogenesis.

The treatment duration of bisphosphonates is still not established. Chapurlat et al reported 20 patients with FD received courses of 180 mg of intravenous pamidronate every 6 months during an average of 39 months (range 18 - 64 months) [12]. In their study, the severity of bone pain and the number of painful sites appeared to be significantly decreased. In addition, the levels of all biochemical markers of bone remodeling were substantially decreased. However, the criteria for discontinuation of the medication remain unclear, including the duration after amelioration of bone pain or that of normalization of the levels of bone markers. So we continue this zoledronate therapy at every 6 months intervals due to high bone turnover and multiple osteofibrous regions.

### Conclusions

We presented the case of an FD patient with persistent elbow pain of 1-year duration due to FD itself and nonunion of humeral fracture, which was ameliorated promptly by intravenous zoledronate therapies. No side effects were evident in this case. Biochemical, radiological improvements and fracture union were observed. This case illustrated the benefit of zoledronate treatment in patients with extensive polyostotic FD and pathological long bone fractures.

## Abbreviations

FD: fibrous dysplasia
ALP: serum alkaline phosphatase
BAP: bone-specific alkaline phosphatase
NTX: urine N-terminal cross-linking telopeptide of type I collagen
